# The Early Events That Initiate β-Amyloid Aggregation in Alzheimer’s Disease

**DOI:** 10.3389/fnagi.2018.00359

**Published:** 2018-11-13

**Authors:** Xingyu Zhang, Zhihui Fu, Lanxia Meng, Mingyang He, Zhentao Zhang

**Affiliations:** Department of Neurology, Renmin Hospital of Wuhan University, Wuhan, China

**Keywords:** Alzheimer’s disease, β-amyloid, aggregation, age, diabetes

## Abstract

Alzheimer’s disease (AD) is characterized by the development of amyloid plaques and neurofibrillary tangles (NFTs) consisting of aggregated β-amyloid (Aβ) and tau, respectively. The amyloid hypothesis has been the predominant framework for research in AD for over two decades. According to this hypothesis, the accumulation of Aβ in the brain is the primary factor initiating the pathogenesis of AD. However, it remains elusive what factors initiate Aβ aggregation. Studies demonstrate that AD has multiple causes, including genetic and environmental factors. Furthermore, genetic factors, many age-related events and pathological conditions such as diabetes, traumatic brain injury (TBI) and aberrant microbiota also affect the aggregation of Aβ. Here we provide an overview of the age-related early events and other pathological processes that precede Aβ aggregation.

## Introduction

Alzheimer’s disease (AD) is the most frequent cause of dementia in the elderly. By 2030, the world’s AD population will reach more than 70 million (McDade and Bateman, 2017). But no effective treatments can prevent, halt, or reverse AD so far. Pathologically, AD is characterized by the assemblies of extracellular β-amyloid (Aβ) plaques and cytoplasmic neurofibrillary tangles (NFTs) consisting of the microtubule-associated protein tau (Braak and Braak, 1991).

Aβ is generated by the sequential proteolytic cleavage of the much larger amyloid precursor protein (APP) by β-secretase and γ-secretase. In contrast, cleavage of APP by α-secretase precludes Aβ formation. The exact physiological function of Aβ remains unknown. In AD brain, Aβ adopts a highly ordered structure known as cross-β spine, or amyloid (Lührs et al., 2005). Many studies have shown a causal relationship between Aβ and the pathogenesis of AD. The NFTs mainly consist of aggregated tau that bears abnormal posttranslational modifications, including hyperphosphorylation, acetylation, ubiquitylation, truncation and so on. Compared to Aβ, tau deposits correlates better with the degree of cognitive impairment (Goedert and Spillantini, 2006). It is believed that tau functions primarily to stabilize microtubes, and its aggregation in AD causes deficits through a loss-of-function mechanism (Morris et al., 2011). However, recent studies have shown that tau may promote or enhance excitatory neurotransmission by modulating the distribution of synaptic activity-related signaling molecules (Morris et al., 2011).

Currently, the predominant framework of AD research is the amyloid hypothesis. According to the amyloid hypothesis (Hardy and Selkoe, 2002), Aβ is the pathological factor that initiates the onset and progression of AD. Thus, Aβ is proposed to be the target of primary prevention trials (McDade and Bateman, 2017). However, what initially triggers the aggregation and accumulation of Aβ in AD is unclear. To stop the disease before it starts, we should find the earlier events that precede Aβ aggregation. This review highlights the relationship between risk factors of AD and Aβ aggregation to bring us closer to a comprehensive understanding of the pathogenesis of AD and prevention potential of early events in AD.

## Assembly of Aβ

Aβ is 40–42 amino acids in length and is formed by proteolytic cleavage of the much larger APP. APP is a transmembrane protein with a single membrane-spanning domain (Glenner and Wong, 1984a,b; Masters et al., 1985), which may have a trophic function (Thornton et al., 2006; Weyer et al., 2011). APP can be cleaved by β-secretase and γ-secretase generating the N terminus and the C terminus of Aβ respectively. During the amyloidogenic process, APP is first cleaved by β-secretase to release the C-terminal fragment (C99), and then C99 is further cleaved by γ-secretase to generate Aβ. In contrast, cleaved by α-secretase precluding Aβ formation. C99 is cleaved at different sites by γ-secretase, resulting in different Aβ profiles (Acx et al., 2014). The major species of Aβ profiles are 40 or 42 amino acids long, and Aβ42 is more aggregation-prone and believed to be the toxic building block of Aβ assemblies.

Aβ adopts a highly ordered structure known as cross-β spine or amyloid (Lührs et al., 2005). The formation of Aβ fibrils can be divided into three phase including nucleation phase, elongation phase and stationary phase (Iadanza et al., 2018). In nucleation phase, oligomeric Aβ forms a nucleus, which can recruit other monomers. As fibrils grow, they can shatter, producing new aggregation-prone species to elongate the fibril. Until nearly all free monomer is converted into a fibrillar form, a variety of insoluble fibrils, oligomers and soluble monomer achieve dynamic balance in the stationary phase. Oligomers are considered to be more pathogenic than mature fiber. However, which Aβ assemblies are most pathogenic is unresolved (Benilova et al., 2012). The fibrils also associate with each other, with other proteins, and with non-proteinaceous factors to form the plaques (Stewart et al., 2017).

Aβ plaques first develop in one or more parts of the basal temporal and orbitofrontal neocortex (Braak and Braak, 1991; Thal et al., 2002; Braak and Del Trecidi, 2015). They were then observed throughout the neocortex, in the hippocampal formation, amygdala, diencephalon and basal ganglia. In severe AD cases, Aβ brain plaque also appears in the mesencephalon, lower brainstem and cerebellar cortex.

Multiple lines of evidence indicate that APP and Aβ contribute causally to the pathogenesis of AD. However, the function of Aβ remains confused. There is evidence that Aβ regulates neuronal and synaptic activity, and that Aβ accumulation in the brain leads to a combination of abnormal network activity and synaptic depression, which can result in excitotoxicity (Palop and Mucke, 2010). Recent studies suggest that Aβ is an antimicrobial peptide, which may play a protective role in innate immunity, and infectious or sterile inflammatory stimuli may drive amyloidosis (Kumar et al., 2016).

## The Amyloid Hypothesis and the Prion Hypothesis

### The Amyloid Hypothesis

In 1992, Hardy and Higgins (1992) postulated that “Aβ … is the causative agent in AD pathology and that NFTs, cell loss, vascular damage and dementia follow as a direct result of this deposition.” This hypothesis has dominated the AD field for more than two decades. A variety of clinical and laboratory evidence supports the hypothesis. The most reliable data supporting the initiator role of Aβ come from genetic studies. The mutations of APP, presenilin-1 (PS1), and PS2, which are involved in Aβ production, cause the autosomal dominant familial AD (fAD; Bettens et al., 2013). Besides, duplication of the APP locus on chromosome 21 in Down syndrome cause age-related dementia with brain parenchymal Aβ deposits (Prasher et al., 1998; Rovelet-Lecrux et al., 2006). Moreover, a rare APP mutation is protective against dementia because it inhibits the production of Aβ and the development of plaques in the brain (Jonsson et al., 2012).

There are also some observations that do not fit easily with the amyloid hypothesis. The main objections can be summed up as the anatomical and temporal discord between Aβ plaque deposition, neuronal death and clinical symptoms in AD (Musiek and Holtzman, 2015). Early neuronal loss regions (entorhinal cortex and hippocampus) are consistent more closely with tau pathology regions than Aβ deposition site (precuneus and frontal lobes), both spatially and temporally (Arriagada et al., 1992; Musiek and Holtzman, 2015). This anatomic disconnection is still not fully explained. However, some studies suggest that the appearance of high-grade cortical tau pathology requires the presence of Aβ aggregation (Price and Morris, 1999; Knopman et al., 2003; Petersen et al., 2006) and tau-mediated toxicity requires trigger from Aβ (West et al., 1994; Gómez-Isla et al., 1996). As for the temporal discrepancy, the neuropathology occurring before symptom onset can be explained as a preclinical AD (Bateman et al., 2012). It is well-accepted that Aβ plays a key role in AD, but it does not exert its effects in a vacuum. The Aβ toxicity involves a complicated network (Musiek and Holtzman, 2015).

### The Prion Hypothesis

The amyloid hypothesis cannot adequately explain the progression of Aβ pathology over a long distance. The recent surge of studies shows the misfolded proteins, such as Aβ and tau, have prion-like properties. Therefore, the prion hypothesis was proposed to explain how amyloid aggregates propagate through anatomically connected brain areas. According to the prion hypothesis, Aβ and tau are similar to prion in the cross-β quaternary structure, the mechanism of self-propagation and cell-to-cell transmission, and the ability to form structurally diverse fibrils (strains; Guo and Lee, 2014). The amyloid formation can be divided into two processes, a slow nucleation phase (the aggregation of the protein into seeds) and a growth phase (the growing fibril break to generate and spread new amyloid seeds; Jucker and Walker, 2013). Also, this seeding process could be homologous or heterologous (Morales et al., 2009), which means oligomers composed of one misfolded protein can promote the polymerization of another protein. This process is termed as “cross-seeding,” which may play an essential and yet uncovered role in the origin of AD.

## What Initiates Aβ Aggregation?

### Genotype of Protein

#### fAD: APP, PS1, Down Syndrome

Although the cases of fAD account for only less than 1% of total AD, the research of fAD helps us to discover the causative gene defects, including APP and PS1. APP gene is located on chromosome 21. It is well-known that APP gene mutations, duplication of its gene or trisomy of chromosome 21 (Down’s syndrome) cause fAD (Prasher et al., 1998). Thirty-nine missense mutations in the APP gene have been described in individuals from Early-onset fAD (Wang Q. et al., 2015), most of which are inside or surrounds the Aβ area. APP mutations either increase total Aβ production or lead to an increased proportion of Aβ42 (Citron et al., 1992; Suzuki et al., 1994). PS1 or PS2 is the catalytic subunit of the γ-secretase protein complex. Mutations in PS1 are the most frequent cause of fAD. The mutations increase the ratio of Aβ42 to Aβ40, which may result from reduced γ-secretase activity (Citron et al., 1997).

#### sAD: ApoE, BIN1 and TREM2

Most cases of AD are sporadic. Inherited forms of the ε4 allele of Apolipoprotein E (ApoE4) was identified as a major genetic risk factor for sAD. Furthermore, the bridging integrator 1 (BIN1 or amphiphysin2) is the second most important genetic susceptibility locus in late-onset AD after ApoE4 (Tan et al., 2013). Recently, rare mutations in triggering receptor expressed on myeloid cells (TREM2) has received much attention, because one of its variants, R47H, is reported to increase the risk for LOAD by 2–3 folds (Guerreiro et al., 2013).

##### ApoE

ApoE is a glycoprotein with a molecular weight of 34.2 kDa (Mahley, 1988), which has three isoforms, ApoE2, ApoE3 and ApoE4, in humans (Mahley and Huang, 2006). ApoE is mainly expressed in brain and liver. Astrocytes and neurons have long been recognized as the primary source of ApoE in the brain (Huang, 2006). The primary role of ApoE is to transport lipids and cholesterol in the body. Besides, ApoE also plays a role in mediating synaptogenesis, synaptic plasticity and neuroinflammation (Holtzman et al., 2012). Corder et al. (1993) reported that subjects with an ApoE ε4 allele had an earlier onset clinical dementia in families with AD. Poirier et al. (1993) further confirmed the association in a case-control study of sporadic AD. This conclusion was supported by a series of other reports (Amouyel et al., 1993; Noguchi et al., 1993; Myers et al., 1996), making the ApoE ε4 allele the most important genetic risk factor for AD. In contrast to APP, PS1 and PS2, the presence of ApoE ε4 is not sufficient to cause the disease. Indeed, despite decades of research, the pathophysiological pathway linking ApoE4 to AD remains unclear. To date, the studies suggest that ApoE4 may promote the pathogenesis of AD via Aβ-dependent and Aβ-independent mechanisms.

###### Aβ-Dependent Mechanisms

ApoE is associated with the formation of amyloid plaques. Lipid-free ApoE3 and ApoE4 can form stable complexes with Aβ peptides. ApoE4 forms complexes with Aβ more efficiently and rapidly than ApoE3 (Huang and Mahley, 2006). Further studies have shown that ApoE binds to residues 12–28 of Aβ and this binding modulates Aβ accumulation, hence affecting disease progression. Peptides that interrupt ApoE/Aβ binding reduced Aβ-related pathology and cognitive improvements in an APP/PS1 transgenic AD mouse model (Liu et al., 2017). On the other hand, lipidated ApoE3 binds Aβ with higher affinity than ApoE4 (Huang and Mahley, 2006), and further studies (Kim et al., 2009) demonstrate that altering ApoE lipidation changes its ability to mediate Aβ clearance or deposition in the brain. Furthermore, recent data describe a novel signal transduction pathway in neurons whereby ApoE activates a non-canonical MAP kinase cascade that enhances APP transcription and amyloid-β synthesis (Huang et al., 2017).

It has been reported that human ApoE regulates Aβ clearance. ApoE2 and ApoE3 clear Aβ more efficiently than ApoE4 (Bales et al., 1999). Also, a C-terminally truncated ApoE4 was found in AD brain, which inefficiently removes Aβ and acts in concert with Aβ to elicit neuronal and behavioral deficits in transgenic mice (Bien-Ly et al., 2011). Overall, ApoE4 may initiate Aβ accumulation through binding with Aβ and decreasing its clearance. Interesting, Wisniewski et al. (1995) isolated Aβ from senile plaques and found that a carboxyl-terminal fragment of ApoE was co-purified. *In vitro*, this fragment could form amyloid-like fibrils. The amyloid-like property of ApoE fragment is reminiscent of the cross-seeding hypothesis. Whether the ApoE fragment initiates Aβ aggregation though cross-seeding needs further investigation.

###### Aβ-Independent Mechanisms

ApoE4 also impairs synaptogenesis and decreases dendritic spine density. This effect is independent of Aβ accumulation (Dumanis et al., 2009; Brodbeck et al., 2011). Besides, the Aβ-independent roles of ApoE4 also include its detrimental effects on neuronal plasticity, aberrant proteolysis that generates neurotoxic fragments, stimulation of Tau phosphorylation and disruption of the cytoskeleton and impairment of mitochondrial function (Huang, 2010).

##### BIN1

Except for ApoE, some studies sought associations between biologically plausible candidate genes and risk of sAD. Among them, BIN1 gene has been identified as the most important genetic risk locus in LOAD after ApoE. Interestingly, although BIN1 mRNA level was found to be increased in AD brains, the protein levels of the longest isoform of BIN1 was decreased, whereas the levels of the shorter BIN1 isoforms were increased (Chapuis et al., 2013; Holler et al., 2014). BIN1 affects AD risk through various pathways, mainly including tau pathology, APP endocytosis/intracellular trafficking, immune/inflammation of the brain, and calcium transients (Tan et al., 2013). Of those, tau pathology is the most studied aspect. BIN1 can interact with tau (Chapuis et al., 2013), and the decline of BIN1 isoform1 promotes the propagation of tau pathology (Calafate et al., 2016). BIN1 is important in the intercellular trafficking of APP, Aβ, ApoE and BACE1 (Tan et al., 2013; Miyagawa et al., 2016; Ubelmann et al., 2017). Miyagawa et al. (2016) found that depletion of BIN1 impaired endosomal trafficking and lysosomal degradation of BACE1, leading to elevated Aβ production. Ubelmann et al. (2017) also found that BACE1 was trapped in tubules of early endosomes and failed to recycle in axons after BIN1 depletion, eventually resulted in increased Aβ production. However, the precise role of BIN1 in the BACE1 recycling remains speculative.

##### TREM2

TREM2 is a transmembrane protein of the immunoglobulin superfamily that is expressed in mononuclear phagocytes, including microglial in brain (Colonna and Wang, 2016). The main function of TREM2 is regulating the microglial phagocytosis and response to inflammatory stimulation. And the individuals carrying the TREM2 variant R47H have an increased risk for AD by 2–3 folds (Guerreiro et al., 2013).

TREM2 binds to anionic ligands including phospholipids, bacterial LPS, sulfatides and DNA (Daws et al., 2003; Cannon et al., 2012; Wang Y. et al., 2015). Recently, Aβ (Zhao et al., 2018), clusterin (CLU; Yeh et al., 2016) and ApoE (Atagi et al., 2015) are also reported to bind the extracellular region of TREM2. The binding with Aβ oligomers mediates Aβ degradation and downstream signaling (Zhao et al., 2018). Additionally, R47H variant impairs Aβ binding (Zhao et al., 2018). The binding with CLU and ApoE also mediate uptake of lipoprotein-Aβ complexes by microglia. Uptake of lipoprotein-Aβ complexes was reduced in individuals carrying a TREM2 AD variant, R62H (Yeh et al., 2016).

TREM2 associated with the adaptor proteins DNAX-activation protein 10 (DAP10) and DAP12. TREM2-DAP10-DAP12 signaling modulates the energetic cellular metabolism by activating the mechanistic target of rapamycin (mTOR; Xing et al., 2015). TREM2-deficient microglia showed a metabolic defect (Ulland et al., 2017), which may result in the microglia ineffectively responding to stressful events, such as Aβ toxicity. Furthermore, some study crossed the TREM2-deficient mice with developed Aβ-plaque-driven mice, and they found microglia of TREM2-deficient mice failed to cluster around Aβ plaque (Wang Y. et al., 2015; Wang et al., 2016; Yuan et al., 2016; Mazaheri et al., 2017; Ulland et al., 2017). The clustering of microglia around Aβ plaque was of significance to limit the Aβ plaque spreading and protect surrounding neurons (Condello et al., 2015; Wang et al., 2016; Yuan et al., 2016). The study further found that the lack of TREM2 increased the Aβ plaque burden in the 5XFAD model of AD (Wang Y. et al., 2015). And the areas not covered by microglia had a high degree of neural dystrophy (Condello et al., 2015). Instead, elevated expression of TREM2 reduced neural dystrophy in the 5XFAD model of AD (Ulland et al., 2017; Lee et al., 2018). However, a TREM2 deficiency in APP/PS1 mice led to a dramatic reduction in Aβ plaque burden (Jay et al., 2015). The different outcomes may due to the use of different mouse models.

TREM2 also modulates the expression of activation markers in disease-associated microglia. TREM2-deficiency failed to upregulate some activation genes (Keren-Shaul et al., 2017). The partial defect of microglial activation may contribute to the development of AD. Recently, TREM2 was found to alter the degradative process in microglia. Zhao et al. (2018) show that TREM2 KO microglia cause defective clearance of Aβ by disrupting proteasome function. Conversely, Lee et al. (2018) reported that lysosomal degradation was involved in Aβ clearance.

Except for the gene mentioned above, there are several genes related to LOAD, such as PLD3, ABCA7, CASS4, CD33, CD2AP, CELF1, CLU, CR1, DSG2, EPHA1, FERMT2, HLA-DRB5-DBR1, INPP5D, MS4A, MEF2C, NME8, PICALM, PTK2B, SLC24H4 RIN3, SORL1, ZCWPW1 (Karch and Goate, 2015). But the relationship with Aβ aggregation is not fully clarified.

### Age-Related Process

Aging is the primary non-genetic risk for sporadic AD, but little is known about how aging affects Aβ generation. Recent summit addressed seven main hallmarks of the basic aging process (Kennedy et al., 2014), including decreased adaptation to stress, loss of proteostasis, stem cell exhaustion, metabolism, macromolecular damage, unfavorable epigenetic and impaired inflammaging. In this review article, we will focus on the mechanism of age-related pathologic process initiating Aβ aggregation.

#### Age-Related Neuronal Stress

Several stress-related signaling pathways are related to AD, such as oxidative stress (Arimon et al., 2015) and nitrosative stress (Guix et al., 2012).

Aging is usually accompanied by accumulation of reactive oxygen species (ROS; Finkel and Holbrook, 2000). Generally, ROS function as messenger and are kept at low level. Excessive amounts of ROS accumulation is defined as oxidative stress, which will damage various cell components. In the brain of AD, ROS production and the level of oxidative stress markers are elevated (Krstic and Knuesel, 2013). Furthermore, lipid peroxidation precedes Aβ deposition (Pratico et al., 2001), suggesting the oxidative stress may be an initiator of Aβ pathology. Further study found that lipid peroxidation product 4-hydroxynonenal (4-HNE) elevated γ-secretase activity and Aβ production, resulting in Aβ and neurodegenerative pathologies in AD (Gwon et al., 2012). Another study also found the β-secretase activity was affected by 4-HNE (Arimon et al., 2015). Oxidative stress was also reported to cause pathogenic PS1 conformational change (Wahlster et al., 2013) and induce Aβ aggregation (Siegel et al., 2007). During the oxidative stress process, superoxide anions react with nitric oxide generating peroxynitrite, which causes nitrosative stress. Peroxynitrite-triggered nitrotyrosination is especially relevant in AD (Smith et al., 1997). Guix et al. (2012) found that the secretion of Aβ is enhanced in an *in vitro* model of neuronal aging. This is associated with an increase in γ-secretase complex formation. Moreover, the age-related nitrative stress promoted the nitrotyrosination of PS1, which is associated with an increased association of the two PS1 fragments, PS1-CTF and PS1-NTF. Further, it raised the Aβ42/Aβ40 ratio.

Repressor element 1-silencing transcription (REST) is also involved in the neuronal stress response. Lu T. et al. (2014) showed that elevated REST levels are associated with the preservation of the ordinary aged people from AD. REST is induced in the aging human brain and regulates a network of genes that mediate cell death, stress resistance and AD pathology. At early stages of AD, REST is lost from the nucleus, resulting in dysregulation of the gene network, including the γ-secretase complex members PS-2 and pen-2, which are implicated in Aβ generation. Interesting, the study also found that aging individuals who harbor substantial AD pathology do not appear to progress to dementia when neuronal REST levels are high.

#### Age-Related Inflammation

Aging is characterized by chronic, systemic, low-grade inflammation (Franceschi et al., 2017). Inflammation is considered to contribute to and exacerbate AD pathology (Gandy and Heppner, 2013; Sudduth et al., 2013; Sarlus and Heneka, 2017). The inflammation in AD includes microglia and astrocytes dysfunction (Xia et al., 2018). One study using viral mimic to stimulated the systemic immune system found the deposition of APP and its proteolytic fragments (Krstic et al., 2012). Some AD-related chronic disease, such as obesity and type 2 diabetes, will lead to the systemic inflammatory condition, which further increases the risk of AD (Takeda et al., 2010; Thaler et al., 2012).

Microglia play the most important role in inflammatory responses in AD. In physiological condition, microglia remove the apoptotic neurons and prune the synaptic connections to keep the normal development of CNS (Paolicelli et al., 2011; Hong et al., 2016). In response to neuropathological insults, including Aβ, microglia alter its morphology and proliferate, express inflammatory markers, phagocytose dead cells and myelin debris, secrete cytokines and neurotrophic factors (Lue et al., 2010). This process is termed as microglial activation. Furthermore, the activation plays a dual role in AD pathogenesis. On the one hand, microglia increase phagocytosis or clearance of Aβ. On the other hand, the persistent production of Aβ leads to the chronic activation of microglia and drive further amyloid deposition (Hickman et al., 2018). Microglia-Aβ interactions lead to NACHT-, LRR- and pyrin (PYD)-domain-containing protein 3 (NLRP3) inflammasome activation (Heneka et al., 2013). After activation, NLRP3 recruits the adaptor protein apoptosis-associated speck-like protein containing a CARD (ASC), triggering ASC helical fibrillar assembly (Lu A. et al., 2014). ASC rapidly bind to Aβ and increase the formation of amyloid-β oligomers and aggregates by a cross-seeding mechanism (Venegas et al., 2017). Under what conditions the microglia play a positive function, and how can we keep the microglia inflammatory response in check and promote clearance of Aβ without accelerating Aβ aggregation remain to be investigated.

Astrocytes also participate in neuroinflammation in AD. Aβ deposition might be a potent trigger of astroglia activation in AD because the cells surround Aβ plaques (Medeiros and LaFerla, 2013). One study found that reducing astrocyte activation in APP/PS1 mice decreased the amyloid levels and ameliorated cognitive and synaptic function (Furman et al., 2012). The result suggested that astrocyte activation may play a deleterious role in AD. However, it has been confirmed that astrocytes can bind and degrade Aβ (Wyss-Coray et al., 2003). But in some mouse models of AD astrocytes show atrophy (Olabarria et al., 2010), which might result in reduced clearance of Aβ. Overall, inflammation plays a complex but important role in AD.

#### Age-Related Disturbances in Proteostasis

Aging is related to a functional decline in protein homeostasis (proteostasis) machinery, contributing to the development of protein misfolding in AD. The proteostasis network (PN) maintains protein homeostasis by controlling the levels of functional proteins and preventing their aggregation. The process is achieved by three branches of the PN, including protein synthesis, the chaperone pathways for the remodeling of misfolded proteins and protein disaggregates, and the protein degradation pathways (Hipp et al., 2014). mTOR pathway acts as a central pathway regulating protein synthesis (Saxton and Sabatini, 2017). Numerous studies have shown that inhibition of mTOR activity could extend lifespan among mammalian species, suggesting mTOR may regulate aging (Antikainen et al., 2017). Activation of mTOR has been recognized as a major event causing the onset of AD (Yates et al., 2013). The activation of mTOR downregulates the autophagy, resulting in reduced clearance of Aβ and elevated Aβ accumulation (Nixon, 2013), and elevates the expressions of β-secretase and γ-secretase by AMPK and IGF-1 pathway (Cai et al., 2015). Aβ aggregates are believed to be detoxified by DAF-16 regulated active aggregation activity that assembles small oligomers into large, less toxic structures and HSF-1 mediated disaggregation and degradation activity (Taylor and Dillin, 2011). Also, DAF-16 and HSF-1 and effector molecules such as kinase mTOR can regulate aging.

### Diabetes

Numerous epidemiological studies suggest that diabetic patients have a significant risk of developing AD (Arvanitakis et al., 2004; Luchsinger et al., 2005; Fukazawa et al., 2013). T2DM increases the risk of dementia by 50%–150% (Strachan et al., 1997; Biessels et al., 2006). However, the underlying mechanisms with clinical relevance remain to be elucidated. Several mechanisms have been proposed, including insulin and insulin-like growth factor (IGF) resistance, glucose toxicity, oxidative stress and mitochondrial dysfunction. Here we focus on the potential mechanisms by which diabetes affects the initiation of Aβ aggregation.

#### Insulin and IGF Resistance

Insulin and IGF signaling is involved in synaptic plasticity, and the organization and function of the brain, playing neuromodulatory and neurotrophic roles (Baglietto-Vargas et al., 2016), and hence may play an essential role in learning and memory. Several studies suggest that insulin and IGF resistance participate in AD pathogenesis (Correia et al., 2011; Cholerton et al., 2013). AD patients have lower brain levels of insulin and insulin receptor (IR), and insulin signaling impairments have been documented in postmortem brain and animal models of AD (Steen et al., 2005; Lester-Coll et al., 2006; Moloney et al., 2010). These abnormalities were associated with increased APP mRNA expression (Steen et al., 2005). A novel study reveals that insulin deficiency alters APP processing by increasing the expression of BACE-1 and accompanied by increased translational upregulation of APP through the PERK-eIF2α phosphorylation pathway (Devi et al., 2012). Another study found that insulin resistance might alter APP processing through autophagy activation (Son et al., 2012). Besides, insulin signaling provides a physiological defense mechanism against Aβ oligomer-induced synapse loss through downregulating oligomer binding sites in neurons (De Felice et al., 2009). Insulin also has multiple anti-amyloidogenic effects on human neuronal cells, including preventing the translocation of the APP intracellular domain fragment into the nucleus, increasing the transcription of anti-amyloidogenic proteins, and increasing the α-secretase-dependent APP-processing pathway (Pandini et al., 2013). On other hand, Aβ oligomers can inhibit insulin signaling via the JNK/TNFα pathway (Bomfim et al., 2012), suggesting a positive feed-forward mechanism.

##### Hyperglycemia

Chronic hyperglycemia characterizes diabetes, and several lines of evidence suggest that hyperglycemia has toxic effects on the brain (Gispen and Biessels, 2000; Kerti et al., 2013). Epidemiological studies show that hyperglycemia individuals had a higher risk of AD and exhibited higher conversion rate from mild cognitive impairment (MCI) to AD, indicating that hyperglycemia might be responsible for AD onset and progression (Crane et al., 2013; Morris et al., 2014). Hyperglycemia may have toxic effects on neurons through several mechanisms, including the direct impact on Aβ, the formation of advanced glycation end products (AGEs; Umegaki, 2014), osmotic insult and oxidative stress.

Hyperglycemia directly raises interstitial fluid (ISF) Aβ levels via altering neuronal activity, which increases Aβ production. K_ATP_ channel impairments mediate hyperglycemia-induced neuronal excitability and increased ISF Aβ (Macauley et al., 2015). Hyperglycemia could also directly inhibit APP protein degradation and enhance Aβ production (Yang et al., 2013). Moreover, hyperglycemia accelerates Aβ aggregation through the formation of AGEs. AGEs are generated by a non-enzymatic reaction of glucose, free amino groups, lipids, and nucleic acids (Singh et al., 2001; Sims-Robinson et al., 2010). The receptors for AGEs (RAGE) are highly expressed in both microglia and neurons and are responsible for the pathological consequences (Lue et al., 2001). Aβ is a RAGE ligand, and Aβ-RAGE interaction exaggerates neuronal stress, accumulation of Aβ, impaired learning and memory and neuroinflammation (Chen et al., 2007). Additionally, AD patients with diabetes (ADD) have higher levels of AGEs than non-diabetic AD individuals (Valente et al., 2010). And RAGE is also demonstrated as a cofactor for Aβ-induced neuronal perturbation in an AD model (Arancio et al., 2004). Oxidative stress is also a key player in diabetes and AD (Moreira, 2012). Oxidative stress stimulates APP gene expression and modulates its processing via modulating γ- and β-secretases (Jolivalt et al., 2010; Oda et al., 2010), which contributes to Aβ aggregation.

##### Cross-Seeding

Islet amyloid polypeptide (IAPP) form β-sheet aggregates in the pancreas in type 2 diabetes (Pillay and Govender, 2013). Interestingly, IAPP deposits are also found in the brain tissue of patients with AD (Jackson et al., 2013). IAPP and Aβ share similar β-sheet secondary structures, and they are 25% identical in amino acid sequence and have a high binding affinity to each other (Andreetto et al., 2010). Emerging evidence indicates that cross-amyloid interactions may play a key role in Aβ aggregation, including the interaction of Aβ-tau (Guo et al., 2006), the Aβ-α-synuclein (Westermark et al., 1996), and the Aβ-IAPP interaction (Andreetto et al., 2010). The potential mechanisms of IAPP-induced AD development include the independently toxic effects, lose the physiological function of soluble IAPP in the brain, and interacting with Aβ (Zhang and Song, 2017). In this part, we will focus on the mechanism of cross-interaction of Aβ and IAPP.

###### *In vitro* Evidence

O’Nuallain et al. (2004) first studied the seeding efficiency between Aβ(1–40) and amyloid fibrils produced from IAPP. They found the IAPP fibrils are poor seeds for Aβ(1–40) elongation. But it is difficult to gauge whether low cross-seeding efficiency might be biologically significant. Later, Yang et al. (2013) found both nucleation and fibrillization of Aβ/hIAPP mixtures (1:1) were slower than pure Aβ or pure hIAPP. And they suggest that the cross-seeding of Aβ and hIAPP was less efficient than homologous seeding of pure Aβ or IAPP. However, a study investigating lipid membranes got different results (Seeliger et al., 2012). They found the aggregation kinetics of Aβ/IAPP mixtures was slower than that of hIAPP, but faster than that of Aβ. Hu et al. (2015) found the cross-seeding of Aβ/IAPP led to the retard of peptide aggregation at the nucleation stage due to structural incompatibility between different amyloid aggregates, and the acceleration at final fibrillation stage due to the formation of similar seed structures as templates for promoting cross-seeding. Another *in vitro* study found that the fibrils of amyloidogenic proteins, including IAPP, functioned as seeds in the Aβ aggregation, and the seeds accelerate the Aβ aggregation pathway. Also, E3, R5, H13, H14 and Q15 of Aβ are common binding regions between the Aβ monomer and the fibrillar seeds including IAPP (Ono et al., 2014). Andreetto et al. (2010) studied the cross- and self-interaction interface of Aβ and IAPP by using membrane-bound peptide arrays and fluorescence titration assays. They identified five short peptide segments of Aβ and IAPP as hot regions of the Aβ-IAPP cross-interaction interface, including Aβ(27–32), Aβ(35–40), Aβ(19–22), IAPP(8–18) and IAPP(22–28), and these peptides also mediate the self-interaction of Aβ and IAPP. They suggested that hetero- and self-association of Aβ and IAPP most likely occur competitively. Overall, the *in vitro* studies investigated the seeding efficiency and the interaction regions of hetero- and homo-seeding, though some results are contradictory. However, amyloid fibrils *in vivo* are functionally different from fibrils grown *in vitro*. So, it is hard to draw biological conclusions from studies only *in vitro*.

###### *In vivo* Evidence

Moreno-Gonzalez et al. (2017) investigated whether IAPP could accelerate Aβ aggregation *in vitro* and *in vivo*. They found that the addition of pre-formed IAPP aggregates to Aβ40 monomers can accelerate the misfolding of Aβ compared with unseeded Aβ monomers *in vitro*, which is consistent with the former studies. Their *in vivo* study found that the transgenic animals expressing human IAPP and mutant APP showed increased Aβ burden in the hippocampus and cortex compared to AD transgenic mice or AD transgenic animals with type 1 diabetes. Additionally, IAPP colocalizes with amyloid plaques in the transgenic mice express hIAPP and mutant APP. Furthermore, injection of pancreatic IAPP aggregates into the APP-mutant mice resulted in more Aβ burden in the cortex and hippocampus and greater memory impairments than untreated animals. Based on these results, the team provided a hypothesis that IAPP aggregates can accelerate the transformation process of Aβ by recruiting the normal soluble protein into the growing aggregates, thereby accelerating or exacerbating the pathological features of AD.

#### Microbes

The microbiota is a newly discovered human organ that weighs about 1.5 kg, and contains approximately 90% of the cells of the human body, with a genetic repertoire that exceeds 100%–200% of the remaining organisms (Qin et al., 2010). The microbes inhabit different locations, including gut, skin, nose and vagina. The gut harbors the highest concentrations of microbiota so far and is the best-studied habitat (Scheperjans, 2016). In recent years, many studies found the association between gut microbes disorders and other disorders including diabetes, obesity, arthritis, allergy, cardiovascular and neurodegeneration diseases. The mechanisms through which gut bacteria influence central process include the neurotransmitters synthesized by gut bacteria, the activation of immune system, the metabolites, such as short-chain fatty acids and amyloid (Sherwin et al., 2018). Except for the microbiota, specific microbes including herpes simplex virus type1 (HSV1), *Chlamydia pneumoniae* and several types of spirochaete which were found in the aging human brain also play a role in the etiology of AD (De Chiara et al., 2012; Balin and Hudson, 2014; Itzhaki, 2014; Miklossy, 2015). In this review article, we will focus on the part of the activation of immune system in the initiation of Aβ.

The microbes of human microbiome can release large amounts of lipopolysaccharides (LPS), which might play a role in the production of proinflammatory cytokines related to the pathogenesis of AD (Pistollato et al., 2016). LPSs may modify gut homeostasis, gut inflammation, and gut permeability (Hufnagel et al., 2013). Additionally, the presence of bacterial LPS or endotoxin-mediated inflammation actively contributes to the potentiated fibrillogenesis of Aβ by stimulating fibril elongation (Asti and Gioglio, 2014) and attenuated amyloid clearance by down-regulating TREM2 (Zhao and Lukiw, 2015). Bacterial amyloid is considered as a pathogen-associated molecular pattern (PAMP) and induces activation of toll-like receptor-2 (TLR2) and other inflammatory mediators including NF-κB, as well as TLR1 and CD14 (Tukel et al., 2010; Nishimori et al., 2012). Besides, the activated inflammatory reaction caused by microbiome species, and their secretory products have shown to intensify the aggregation of amyloids into senile plaque lesions (Smith et al., 1996; Zhao et al., 2018).

Allen (2016) provided a novel hypothesis about the production of Aβ induced by spirochetes. They found spirochetes and innate immune system activity in the brains of AD patients. Additionally, they suggested that the innate immune system first responder TLR2 and its major pathway (MyD88) activates the secretases which generate Aβ. Interestingly, Aβ has been shown to be antimicrobial (Soscia et al., 2010). Therefore, they suggested that Aβ is generated for the purpose to rid the body of the spirochetal parasites, and the damages of tissue, as well as the neuronal circuits, are the adverse reactions. Kumar et al. (2016) gave a more detailed explanation of the antimicrobial role of Aβ. They found a rapid seeding and accelerated Aβ deposition after Salmonella Typhimurium bacteria infections in 5XFAD mice. And they showed that Aβ fibrils mediated adhesion inhibition and agglutination activities against Candida. This novel perspective deepens our understanding of the enigmatic role of Aβ in the etiology of AD.

#### Traumatic Brain Injury

Traumatic brain injury (TBI) is an universal health and socioeconomic problem. The risk of AD is increased in moderate and severe head injury for 2.3 and 4.5 times, respectively (Plassman et al., 2000). Besides, a growing number of epidemiological studies have considered TBI as one of the most potent risk factors for AD (Molgaard et al., 1990; O’Meara et al., 1997; Guo et al., 2000; Fleminger et al., 2003). However, two recent studies found that a history of TBI was not associated with AD or the Aβ deposition (Crane et al., 2016; Weiner et al., 2017). The conflicting result may due to the difference in severity and frequency of TBI, which may result in different neuropathologic outcomes.

Numerous studies have shown that TBI can trigger rapid and insidiously progressive AD-like pathological process, such as the production and accumulation of Aβ (Johnson et al., 2010). Up to 30% of patients who die from TBI have the Aβ plaques in their brain (Roberts et al., 1991, 1994). But the mechanism by which TBI induce Aβ accumulation is still obscure. The most common pathologies of TBI is diffuse axonal injury, which causes an accumulation of proteins in the axon, including APP (Gentleman et al., 1993; Gorrie et al., 2002). Also, PS-1 and BACE1 were found in injured axons after TBI (Uryu et al., 2007; Chen et al., 2009). Furthermore, high APP production following TBI may increase β-secretase processing and Aβ genesis (Lou et al., 2017), due to the saturation of normal α-secretase processing pathway (Gentleman et al., 1993; Graham et al., 1996). TBI also induces Aβ genesis via oxidative-stress-mediated upregulation of BACE1 (Tamagno et al., 2005; Guglielmotto et al., 2009). TBI is accompanied by hypoperfusion, vascular dysfunction and ischemia (Ramos-Cejudo et al., 2018), which may play an important role in Aβ deposition. It has been shown that transient hypoxia elevated plasma Aβ42 levels (Gren et al., 2016); reduced blood flow activated β-secretase and γ-secretase (Pluta et al., 2013); metabolic acidosis after TBI could potentially contribute to Aβ accumulation due to the fact that Aβ is prone to aggregation in a pH-dependent manner (Acharya et al., 2016). S100A9-driven amyloid-neuroinflammatory cascade may be also involved in the accumulation of Aβ. S100A9 is an amyloidogenic protein associated with inflammation, which was found to form amyloid plaques itself in TBI (Wang et al., 2018) and AD (Shepherd et al., 2006). Recently, S100A9 was found abundant in TBI human brain tissue compared to Aβ and contributed to Aβ plaque formation (Wang et al., 2018). Another study also found S100A9 also co-aggregated with both Aβ40 and Aβ42 and promoted their amyloid deposition (Wang et al., 2014). How S100A9 interact with Aβ and whether aggregation of S100A9 could serve as seeds to accelerate aggregation of Aβ need a deep investigation.

### Others

Beside processes mentioned above, many factors may influence the production and accumulation of Aβ. For example, dietary fats may affect cerebrovascular integrity and alter Aβ kinetics across the blood-brain barrier (Takechi et al., 2010). Sex hormones also have effects on Aβ pathology (Grimm et al., 2016). Women exhibit a greater vulnerability to AD (Mielke et al., 2014) and a more striking Aβ deposition compared to men (Corder et al., 2004). Additionally, olfactory impairment subjects have more Aβ accumulation than normal people (Vassilaki et al., 2017).

## Conclusions

A wealth of studies supports the amyloid hypothesis that Aβ is the initiator of a complex network of pathologic changes in the brain. And many earlier events precede Aβ aggregation. The best way to eliminate the Aβ pathology is to stop it from taking hold in the first place. Although much has been learned, many important questions remain. How do the early events initiate Aβ aggregation? How can we prevent it? What is the target point? When should measures be taken? How to explain the pathogenesis of AD-like dementias without Aβ, and how to avoid it? The answers to these questions might bring us to find safe and effective treatments for AD.

## Author Contributions

XZ, ZF, LM, MH and ZZ prepared the manuscript.

## Conflict of Interest Statement

The authors declare that the research was conducted in the absence of any commercial or financial relationships that could be construed as a potential conflict of interest.
